# The role of brown adipose tissue in branched-chain amino acid clearance in people

**DOI:** 10.1016/j.isci.2024.110559

**Published:** 2024-07-20

**Authors:** Yasser G. Abdelhafez, Guobao Wang, Siqi Li, Vanessa Pellegrinelli, Abhijit J. Chaudhari, Anthony Ramirez, Fatma Sen, Antonio Vidal-Puig, Labros S. Sidossis, Samuel Klein, Ramsey D. Badawi, Maria Chondronikola

**Affiliations:** 1 Department of Radiology, University of California Davis, Sacramento, CA 95817, USA; 2 Nuclear Medicine Unit, South Egypt Cancer Institute, Assiut University, El Fateh 71111, Egypt; 3 Institute of Metabolic Science-Metabolic Research Laboratories, Medical Research Council Metabolic Diseases Unit, University of Cambridge, Cambridge CB2 0QQ, UK; 4 Department of Nutrition, University of California Davis, Davis, CA 95616, USA; 5 Department of Kinesiology and Health, Rutgers University, New Brunswick, NJ 08901, USA; 6 Center for Human Nutrition, Washington University School of Medicine, Saint Louis, MO 63110, USA; 7 Department of Nutrition and Dietetics, Harokopio University of Athens, 17778 Athens, Greece

**Keywords:** Human genetics, Human metabolism, Cancer

## Abstract

Brown adipose tissue (BAT) in rodents appears to be an important tissue for the clearance of plasma branched-chain amino acids (BCAAs) contributing to improved metabolic health. However, the role of human BAT in plasma BCAA clearance is poorly understood. Here, we evaluate patients with prostate cancer who underwent positron emission tomography-computed tomography imaging after an injection of ^18^F-fluciclovine (L-leucine analog). Supraclavicular adipose tissue (AT; primary location of human BAT) has a higher net uptake rate for ^18^F-fluciclovine compared to subcutaneous abdominal and upper chest AT. Supraclavicular AT ^18^F-fluciclovine net uptake rate is lower in patients with obesity and type 2 diabetes. Finally, the expression of genes involved in BCAA catabolism is higher in the supraclavicular AT of healthy people with high BAT volume compared to those with low BAT volume. These findings support the notion that BAT can potentially function as a metabolic sink for plasma BCAA clearance in people.

## Highlights

• Human BAT clears fluciclovine (BCAA analog for PET imaging) faster than abdominal AT
• BCAA uptake in BAT is lower in people with obesity and type 2 diabetes
• BCAA catabolic genes expression is higher in the BAT of people with high BAT volume

## Graphical abstract

**Figure:**
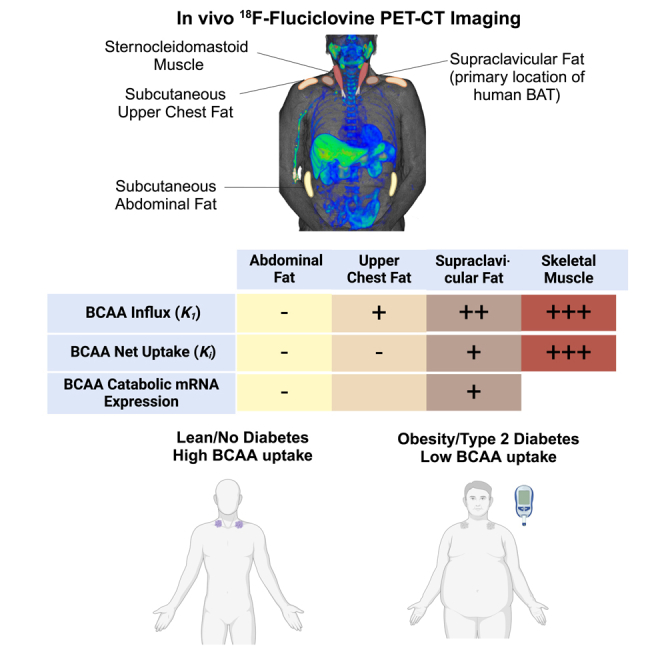

## Introduction

Branched-chain amino acids (BCAAs: leucine, valine, and isoleucine) are essential amino acids that have a central carbon atom with an aliphatic side chain of three or more carbons (branch) attached to it. Apart from their role as structural building blocks of proteins, BCAA act as signaling molecules regulating numerous important metabolic processes (e.g., protein synthesis, insulin secretion, etc.).^1^ High plasma BCAA concentration has been associated with insulin resistance and is thought to contribute to the pathogenesis of type 2 diabetes (T2D)^2^^,^^3^^,^^4^^,^^5^ and other cardiometabolic conditions.^6^^,^^7^ Although there has been intense scientific interest in understanding how BCAAs affect metabolic health, their mechanism of action is not completely understood.

Brown adipose tissue (BAT) has been proposed as a potential target for the prevention of cardiometabolic disease, due to its high capacity for substrate oxidation to produce heat.^8^ Although circulating lipids and glucose are the major substrates for BAT thermogenesis,^9^ more recent data suggest that BAT is also an avid consumer of plasma BCAA.^10^^,^^11^^,^^12^^,^^13^ Increased BCAA catabolism in BAT has been shown to increase insulin sensitivity and reduce adiposity in rodents.^10^ Early evidence from clinical investigations also supports the link between BAT and BCCA metabolism in people.^10^^,^^14^ However, these studies lack tissue-specific evidence supporting the direct involvement of BAT in the regulation of BCAA metabolism and its quantitative contribution in plasma BCCA clearance.

To this end, we conducted a proof-of-concept study aiming to determine whether human BAT has higher uptake rate for BCAA compared to other subcutaneous adipose tissue depots. We hypothesized that supraclavicular adipose tissue (the primary location of human BAT^15^) would demonstrate a higher BCAA uptake rate and a higher expression of genes involved in BCAA catabolism compared to subcutaneous abdominal and/or upper chest white adipose tissue. ^18^F-fluciclovine is a synthetic L-leucine analog positron emission tomography-computed tomography (PET-CT) radiotracer^16^ that has been developed and clinically used as cancer imaging agent.^17^ To this end, ^18^F-fluciclovine total-body PET/CT dynamic scans, conducted for clinical purposes, were analyzed using tracer kinetic modeling to evaluate BCAA kinetics in the supraclavicular, subcutaneous upper chest and abdominal adipose tissue. Furthermore, we assessed the expression of key genes involved in BCAA catabolism in supraclavicular and subcutaneous abdominal adipose tissue in a distinct cohort of healthy adults with overweight/obesity.

## Results

### Participant characteristics

We identified 36 adult male patients with histopathologically proven prostate adenocarcinoma who underwent ^18^F-fluciclovine PET-CT imaging as part of their standard clinical care. The study participants were older, mostly white, and non-Hispanic (Table 1). The body mass index (BMI) of the participants ranged from the normal to the obese BMI category. The prevalence of T2D diagnosis in the study population was 13.8% (*n* = 5).

**Table 1:** Patient demographic and anthropometric characteristics

Parameters	*n* = 36
Age (years)	72.2 ± 8.3
Race (White/unknown)	33/3
Ethnicity (non-Hispanic/Asian/unknown)	33/1/2
BMI (kg/m^2^)	27.9 ± 5.2
Weight (kg)	88.4 ± 19.3
Height (cm)	178 ± 8

Data are means ± SD. Related also to Table S1.

### Tissue-specific metabolic assessment using ^18^F-fluciclovine PET-CT imaging

**Figure 1:**
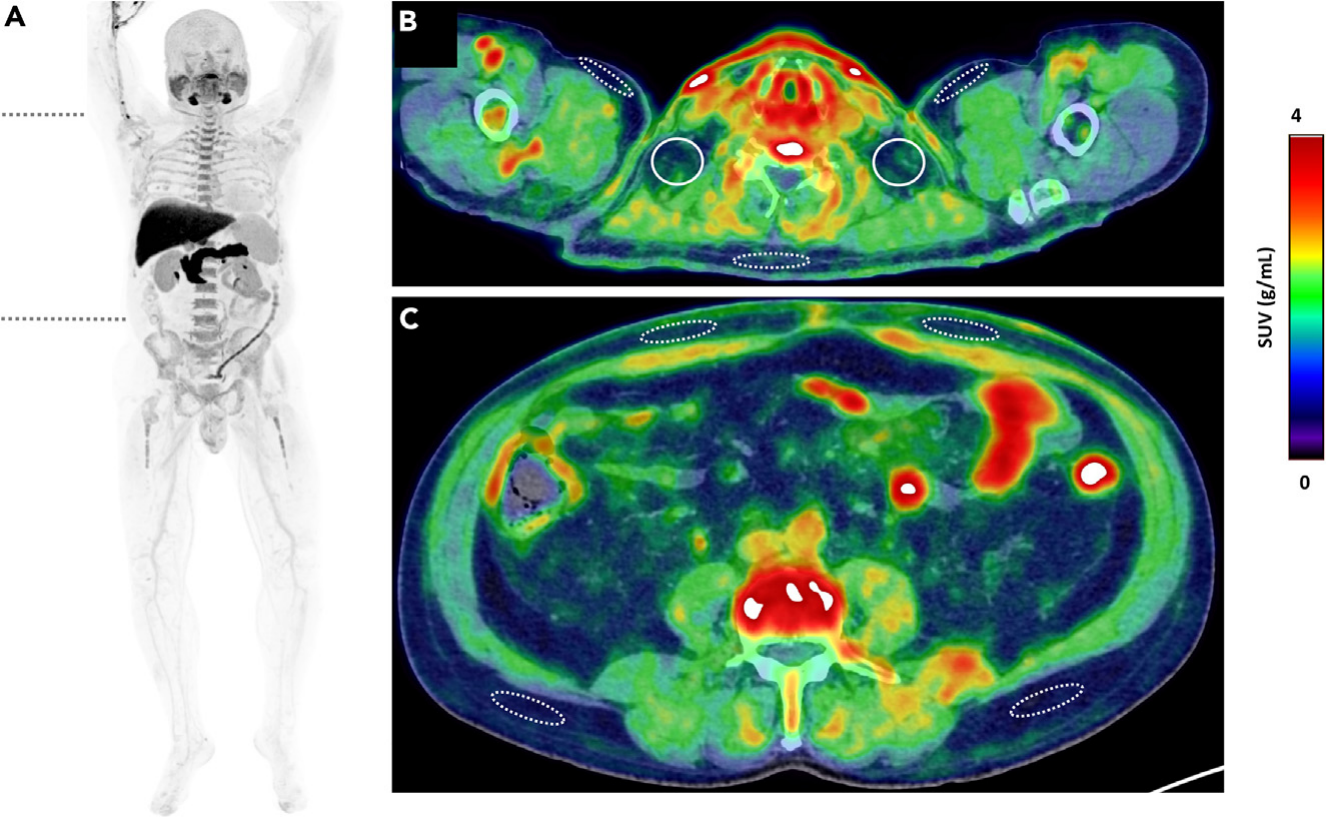
Normal ^18^F-fluciclovine biodistribution Maximum intensity projection image of a 61-year-old man (183 cm, 98 kg) with biochemical recurrence of prostate cancer, demonstrating physiologic ^18^F-fluciclovine uptake, being most prominent in the pancreas, liver, salivary glands, and bone marrow. The two transverse dashed lines on (A) point to the level of cross-sectional axial fused PET/CT images (B) and (C), demonstrating supraclavicular adipose tissue depot (solid circles, B), upper chest subcutaneous adipose tissue (dashed ellipses, B), and abdominal subcutaneous adipose tissue depots at various locations opposite third lumbar vertebra (dashed ellipses, C). Visually, the supraclavicular regions show subtle higher ^18^F-fluciclovine uptake compared to other regions shown in (B) and (C). The difference was more evident on quantification.

**Figure 2:**
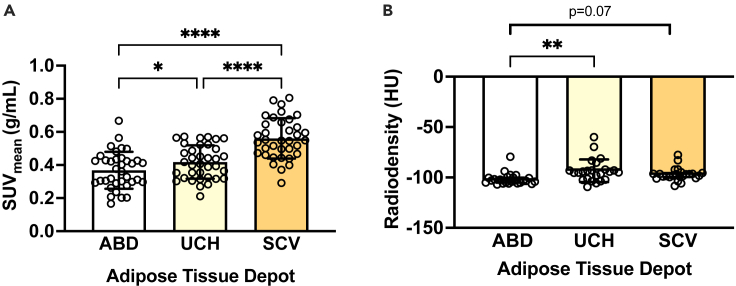
Tissue-specific^18^F-fluciclovine uptake assessment and CT density (A) SUV_mean_ and (B) radiodensity of various tissues assessed by using ^18^F-fluciclovine positron emission tomography-computed tomography (*n* = 36). Data are presented as mean ± SD (for normally distributed data) or median (interquartile range) for skewed data. *p* values were determined by using analysis of variance (for normally distributed data) with Tukey correction for multiple comparisons or Friedman test (for skewed data) with Dunn correction for multiple comparisons. ABD,: abdominal; CT, computed tomography; HU, Hounsfield units; SCV, supraclavicular; UCH, upper chest; SUV, standardized uptake value. ∗*p* < 0.05, ∗∗*p* < 0.01, and ∗∗∗∗*p* < 0.0001.

To evaluate whether human BAT has a high capacity for BCAA uptake, we first assessed the mean standardized uptake value for ^18^F-fluciclovine (standardized uptake value [SUV_mean_]: index of ^18^F-fluciclovine uptake determined using static PET imaging of a single time frame 4–14 min post-injection) in supraclavicular adipose tissue (the primary location of human BAT^15^), the upper chest (upper body subcutaneous adipose tissue depot), and subcutaneous abdominal adipose tissue (classical white adipose tissue depot^18^) (Figures 1A–1C). The supraclavicular adipose tissue had the highest SUV_mean_ for ^18^F-fluciclovine compared to the subcutaneous upper chest and abdominal adipose tissue (Figure 2A). Additionally, the SUV_mean_ for ^18^F-fluciclovine was higher in the upper chest compared to the abdominal adipose tissue (Figure 2A). The radiodensity (which correlates negatively with lipid content and positively with arterial blood volume^19^) of the three adipose tissue depots was also assessed using the CT imaging data. Tissue radiodensity was (marginally) higher in the supraclavicular and upper chest adipose tissue compared to the abdominal adipose tissue, while it was similar between the upper chest and the supraclavicular adipose tissue (Figure 2B).

**Figure 3:**
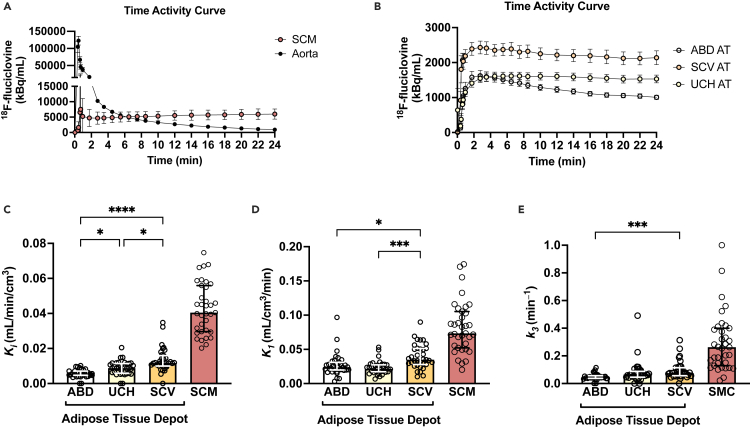
^18^F-fluciclovine kinetics in the different adipose tissue depots using PET imaging (A and B) PET time-activity curves over the 24 min of acquisition after ^18^F-fluciclovine injection in (A) blood pool in the ascending aorta (as input function) and sternocleidomastoid muscle as reference tissue as well as (B) in the adipose tissue depots of interest (*n* = 35). Each time point represents a mid-frame value. Data are means ± SEM. (C) Tracer delivery rate (*K*_*1*_: index of tissue perfusion) in the different adipose tissue depots (*n* = 35). (D) Net uptake rate (*K*_*i*_) for ^18^F-fluciclovine in the different adipose tissue depots (*n* = 35). (E) Flux rate from interstitial to intracellular compartment (*k3*) for ^18^F-fluciclovine in the different adipose tissue depots (*n* = 35). Parametric analysis was not performed for one participant due to lack of imaging data for the first 4 min after injection. Data are median and interquartile range. *p* values were determined by using the Friedman test with Dunn correction for multiple comparisons. ABD, abdominal; SCM, sternocleidomastoid muscle; SCV, supraclavicular; UCH, upper chest; PET, positron emission tomography. ∗*p* < 0.05, ∗∗∗*p* < 0.001, and ∗∗∗∗*p* < 0.0001. Related also to Figures S1–S3.

A two-tissue compartmental model (Figure S1) has been implemented to quantitatively describe ^18^F-fluciclovine kinetics in the three adipose tissue depots of interest. Considering that skeletal muscle is a major tissue responsible for the BCAA plasma clearance,^11^ ^18^F-fluciclovine kinetics were also assessed in the sternocleidomastoid muscle as a reference tissue. Figures 3A–3B present the time-activity curves for ^18^F-fluciclovine in aorta, sternocleidomastoid muscle, and the three adipose tissue depots of interest. Supraclavicular adipose tissue had the highest net uptake rate *Ki* (which provides an assessment of the clearance of ^18^F-fluciclovine from blood to tissue) compared to the subcutaneous abdominal and upper chest adipose tissue (Figure 3C). Upper chest adipose tissue had a higher net uptake rate *Ki* for ^18^F-fluciclovine compared to the abdominal adipose tissue. *K1* (which provides an assessment of tracer delivery rate to the tissue and partially reflects tissue perfusion, Figure 3D) and *k3* (which provides an assessment of the flux rate from interstitial to intracellular compartment, Figure 3E) parameters were also higher in supraclavicular compared to the abdominal adipose tissue. Additionally, the supraclavicular adipose tissue had a higher *K1* compared to the upper chest adipose tissue, but the *k3* was similar between the two tissues (Figures 3D and 3E). Skeletal muscle had higher ^18^F-fluciclovine delivery rate *K1*, net uptake rate *Ki*, and *k3* flux rate from interstitial to intracellular compartment compared to all adipose tissue depots assessed (Figures 3C–3E). Supraclavicular adipose tissue SUV_mean_ and net uptake rate *Ki* were not different among participants imaged during the four different seasons (Figures S2A and S2B). Subsequently, we investigated the interrelationships between supraclavicular adipose tissue ^18^F-fluciclovine kinetic parameters and radiodensity. Supraclavicular adipose tissue ^18^F-fluciclovine net uptake rate *Ki* positively correlated with radiodensity, SUV_mean_, and ^18^F-fluciclovine delivery rate *K1* (Figures S3A–S3C). Supraclavicular adipose tissue SUV_mean_ for ^18^F-fluciclovine correlated with tissue radiodensity, while tissue radiodensity marginally and weakly correlated with ^18^F-fluciclovine delivery rate *K1* (Figures S3D and S3E).

### Plasma BCAA clearance as a biomarker of adiposity and metabolic health

**Figure 4:**
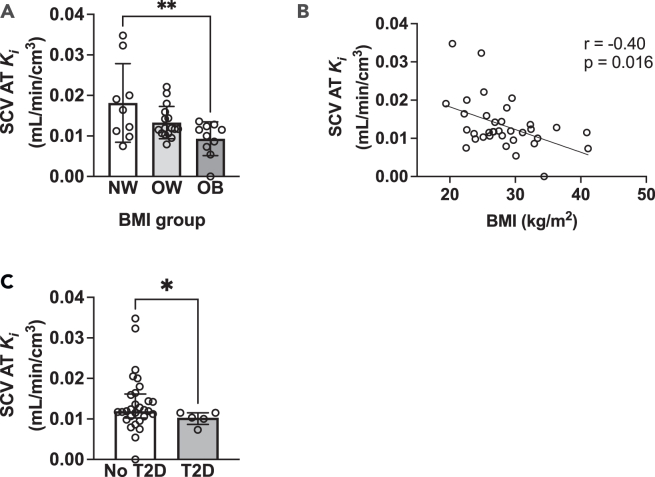
^18^F-fluciclovine net uptake rate in supraclavicular adipose tissue as a marker of adiposity and metabolic health (A) SCV adipose tissue net uptake rate for ^18^F-fluciclovine in patients in the normal (*n* = 9), overweight (*n* = 16), and obese (*n* = 10) BMI category. *p* values determined by using one-way analysis of variance with Tukey correction for multiple comparisons. (B) Correlation between SCV adipose tissue net uptake rate for ^18^F-fluciclovine and BMI (*n* = 35). Spearman’s rho was used to evaluate the correlation between the different variables of interest. (C) SCV adipose tissue net uptake rate for ^18^F-fluciclovine in patients with (*n* = 5) and without diagnosis of T2D (*n* = 29). The medical record of one of the participants was not available. *p* value was determined by using Mann-Whitney U test. Data are means ± SD for normally distributed data or median (interquartile range) for skewed data. AT, adipose tissue; BMI, body mass index; *K*_*i*_, net uptake rate reflecting clearance from blood; NW, normal weight; OB, obesity; OW, overweight; SCV, supraclavicular; T2D, type 2 diabetes. ∗*p* < 0.05 and ∗∗*p* < 0.01.

Considering that increased plasma BCAA catabolism in BAT has been implicated in the development of adiposity and insulin resistance,^10^ we evaluated the link between ^18^F-fluciclovine net uptake rate *Ki* in the supraclavicular adipose tissue with adiposity and T2D. There was a progressive decrease of ^18^F-fluciclovine net uptake rate *Ki* in the supraclavicular adipose tissue from the normal to the obese BMI category (Figure 4A), while BMI was inversely correlated with the ^18^F-fluciclovine net uptake rate in the supraclavicular adipose tissue (Figure 4B). Further, patients with diagnosis of T2D had significantly lower supraclavicular adipose tissue ^18^F-fluciclovine net uptake rate compared to participants without diagnosis of T2D (Figure 4C). *Post hoc* exploration of the study results revealed that BMI was negatively correlated with net uptake rate *Ki* for ^18^F-fluciclovine in the upper chest adipose tissue (r = −0.43, *p* = 0.01) and the ^18^F-fluciclovine delivery rate *K1* in the abdominal adipose tissue (r = −0.43, *p* = 0.011). BMI was not correlated with the net uptake rate for ^18^F-fluciclovine in abdominal adipose tissue (r = −0.17, *p* = 0.33) and skeletal muscle (r = −0.075, *p* = 0.67). Similarly, BMI was not correlated with the ^18^F-fluciclovine delivery rate *K1* in upper chest adipose tissue (r = 0.13, *p* = 0.46), supraclavicular adipose tissue (r = −0.20, *p* = 0.25), or skeletal muscle (r = 0.16, *p* = 0.35).

### Expression of genes involved in BCAA metabolism in the supraclavicular and subcutaneous abdominal adipose tissue

**Figure 5:**
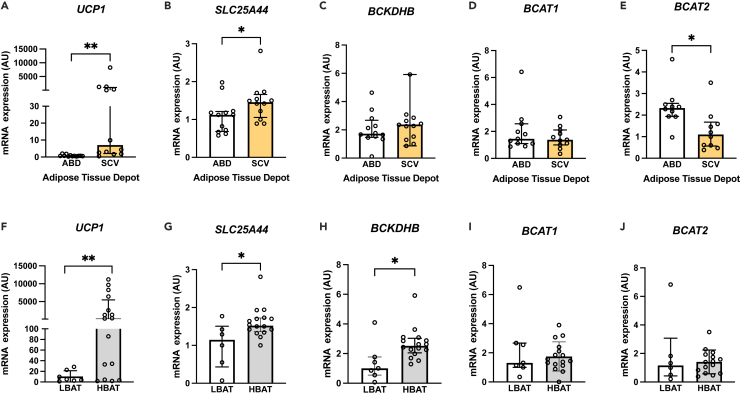
Expression of genes involved in BCAA metabolism: adipose tissue depot-specific variation and BAT status (A–E) Expression of genes involved in BCAA metabolism and thermogenesis in paired supraclavicular and abdominal adipose tissue samples (*n* = 10–12). *p* values were determined by using the Wilcoxon signed-rank test. (F–J) Expression of genes involved in BCAA catabolism and thermogenesis in the supraclavicular adipose tissue depot of people with low (n = 6–7) and high (*n* = 15–16) BAT volume (LBAT and HBAT, respectively). Data are median and interquartile range. *p* values were determined by using the Mann-Whitney test. The smaller number of observations in (D), (E), and (J) are due to limited sample availability. BAT, brown adipose tissue; BCAA, branched-chain amino acids; BCAT1/2, branched-chain amino acid aminotransferase 1 or 2; BCKDHB, branched-chain keto acid dehydrogenase E1 subunit beta; HBAT, group of participants with brown adipose tissue volume equal to or more than 20 mL; LBAT, group of participants with brown adipose tissue volume less than 20 mL; SLC25A44, solute carrier family 25 member 44; UCP1, uncoupling protein 1. ∗*p* < 0.05 and ∗∗*p* < 0.01. Related to Tables S1 and S2.

To further assess the role of human BAT in BCAA metabolism, we evaluated the expression of key genes involved in BCAA mitochondrial uptake and breakdown in supraclavicular and subcutaneous abdominal adipose tissue in a different cohort of healthy men and women with overweight/obesity (Table S1). The expression of *Uncoupling Protein 1* (*UCP1*, which encodes the major thermogenic protein)^9^ and S*olute Carrier Family 25 Member 44* (*SLC24A44*, which encodes a key mitochondrial carrier protein involved in the transport of BCAA from the cytosol into the mitochondria^10^) were higher in the supraclavicular compared to the abdominal adipose tissue (Figures 5A and 5B). The expression of *Branched-Chain Keto Acid Dehydrogenase E1 Subunit Beta (BCKDHB)*, which encodes a subunit of a multienzyme mitochondrial complex involved in the catabolism of BCAA,^10^ was about 50% (but not statistically significantly higher) in the supraclavicular compared to the subcutaneous abdominal adipose tissue (Figure 5C). *Branched-chain-amino-acid transaminase 1* (*BCAT1,* which encodes the cytosolic enzyme that converts BCAA to branched-chain keto acids in the cytosol^20^) expression was similar in the supraclavicular and abdominal adipose tissue, while *branched-chain-amino-acid transaminase 2* (*BCAT2*, which encodes the enzyme that converts BCAA to branched-chain keto acids in the mitochondria^20^) expression was higher in the abdominal adipose tissue compared to the supraclavicular adipose tissue depot (Figures 5D and 5E). Finally, we compared the expression of the previously mentioned genes in the supraclavicular adipose tissue of participants with high 2-Deoxy-2-[^18^F]-fluorodeoxyglucose PET-detectable BAT volume ≥20 mL (HBAT) and participants with low BAT volume <20 mL (LBAT).^21^^,^^22^^,^^23^ We observed no statistically significant differences in terms of age and anthropometric and demographic characteristics between the two groups (Table S1). The expression of the *UCP1* gene along with the expression of *BCKDHB* and *SLC24A44* was higher in the supraclavicular adipose tissue of HBAT group compared to the LBAT group (Figures 5F–5H). The *BCAT1* and *BCAT2* gene expression was similar in the two groups (Figures 5I and 5J). *UCP1* expression in the supraclavicular adipose tissue depot was positively correlated with *SLC24A44* (r = 0.38, *p* = 0.07) and *BCKDHB* expression (r = 0.44, *p* = 0.04), but not with the expression of *BCAT1* (r = 0.17, *p* = 0.44) and *BCAT2* (r = −0.04, *p* = 0.86).

## Discussion

Increased plasma BCAA concentrations have been linked to the pathogenesis of T2D^2^^,^^3^^,^^4^; hence understanding the mechanism(s) regulating BCAA metabolism can have important implications for the development of effective strategies for the prevention of T2D. Here, we report that the supraclavicular adipose tissue—the primary location of human BAT^15^—can more effectively remove BCAA from the circulation compared to other subcutaneous adipose tissue depots investigated in this study. The higher BCAA net uptake rate in supraclavicular adipose tissue was attributable both to the increased perfusion rate of the tissue and to its intrinsic metabolic characteristics. The supraclavicular adipose tissue BCAA net uptake rate was inversely correlated with tissue radiodensity, while it was lower among people with increased adiposity and T2D. Consistent with these findings, transcriptional analysis of supraclavicular adipose tissue and subcutaneous adipose samples, collected from a different cohort of participants, further indicates that BAT is a tissue with a high capacity for BCAA catabolism compared to subcutaneous abdominal adipose tissue. Although BAT *per se* may have a higher capacity for plasma BCAA clearance compared to other subcutaneous adipose tissue depots, it is unlikely to have a major effect in regulating plasma BCAA concentrations due to its small measurable volume in people.

The findings of this study support the notion that human BAT may be an adipose tissue depot with increased ability to remove BCAA from the circulation compared to upper chest and subcutaneous abdominal adipose tissue depots. The analysis performed using dynamic ^18^F-fluciclovine PET-CT imaging data indicates that the supraclavicular adipose tissue has the highest net uptake rate for ^18^F-fluciclovine (*Ki*) compared to the subcutaneous upper chest and abdominal adipose tissue. The transcriptional data from a different cohort of healthy participants are consistent with ^18^F-fluciclovine kinetic data. The expression of the *SLC25A44* (a key mitochondrial carrier protein involved in the transport of BCAA from the cytosol into the mitochondria^10^) and *UCP1* was higher in the supraclavicular compared to abdominal adipose tissue. Similarly, the expression of genes involved in thermogenesis and BCAA catabolism (i.e., *SLC25A44* and *BCKDHB*), but not *BCAT2*, was higher in the supraclavicular adipose tissue of participants with high BAT volume compared to those with low BAT volume. The expression of *BCAT2* was higher in the abdominal adipose tissue compared to the supraclavicular adipose tissue. *BCAT2* encodes the mitochondrial enzyme that catalyzes the reversible transamination of BCAA to branch-chained keto acids (first step in BCAA oxidation pathway), whereas *BCKDHB* encodes the subunit of a multienzyme mitochondrial complex that catalyzes the oxidative decarboxylation branched-chained ketoacids (the second and rate-limiting step in BCAA catabolism).^20^ Interestingly, *UCP1* expression in supraclavicular adipose tissue was positively correlated with *SLC25A44* and *BCKDHB* gene expression suggesting that BCKDH and SLC24A44 may be involved in the regulation of thermogenesis in BAT as it has been previously reported in rodents.^24^ Although decreased BCAT2 activity in white adipose tissue rodents has been associated with the browning of white adipose tissue and prevention of obesity in rodents,^24^ we found no relationship between *BCAT2* and *UCP1* expression in the supraclavicular adipose tissue depot.

To evaluate the physiological mechanisms mediating the high net uptake rate (*K*_*i*_) for ^18^F-fluciclovine observed in the supraclavicular adipose tissue compared to the other adipose tissue depots investigated, we assessed the *K*_*1*_ tracer delivery rate (marker of perfusion) and *k*_*3*_ flux rate from interstitial to intracellular compartment for target tissue. The supraclavicular adipose tissue has a higher *K*_*1*_ tracer delivery rate and *k*_*3*_ flux rate from interstitial to intracellular compartment compared to the abdominal adipose tissue, suggesting that both intrinsic tissue-specific metabolic characteristics and higher perfusion underlie the higher net uptake (*K*_*i*_) for ^18^F-fluciclovine reported in the supraclavicular adipose tissue compared the abdominal adipose tissue. Conversely, supraclavicular adipose tissue has a higher *K*_*1*_ tracer delivery rate compared to the upper chest adipose tissue, but a similar ^18^F-fluciclovine flux rate *k*_*3*_ from interstitial to intracellular compartment, suggesting that increased perfusion may explain the higher net uptake rate detected in the supraclavicular adipose tissue depot compared to the upper chest adipose tissue.

The results of this study advance the current understanding on the role of BAT in the regulation of BCAA metabolism. BAT in rodents is thought to be responsible for ∼20% of whole-body BCAA oxidation in room temperature conditions (mild cold for rodents) suggesting that BAT is the organ with the second highest contribution to whole-body BCAA oxidation after skeletal muscle^11^; hence its contribution in thermoneutrality is estimated to be smaller. Consistent with these findings, Yoneshiro et al. reported that plasma BCAA catabolism in BAT is implicated in the BAT thermogenesis and the development of adiposity and insulin resistance.^10^ The role of human BAT in BCAA metabolism has been studied only indirectly. Namely, acute cold exposure has been shown to decrease circulating BCAA levels in lean men with high BAT activity,^10^ while the ratio of fasting BCAA to total amino acid concentration has been correlated with total hemoglobin concentration in the supraclavicular adipose tissue (marker of BAT vascular density) in thermoneutrality (∼24°C).^14^ Our findings are consistent with previous studies in rodents regarding the high capacity of BAT for BCAA uptake; hence we expect that the absolute contribution of BAT in the regulation of whole-body plasma BCAA clearance will be small considering that skeletal muscle has approximately a 3-fold higher uptake rate per volume of tissue compared to supraclavicular adipose tissue and is several fold larger in volume. In this study, we were unable to assess the total contribution of BAT in plasma BCAA clearance as the patients did not perform ^18^F-FDG PET-CT imaging that would enable us to quantify BAT volume,^25^ and arterial blood samples were not collected to allow the absolute quantification of leucine uptake. However, our results provide some estimates on the contribution of BAT in BCAA clearance in the absence of cold stimulation. Assuming BAT volume is ∼100 cm^3^ and a net uptake rate for ^18^F-fluciclovine in the supraclavicular depot of 0.012 mL/min per cm^3^ of tissue, we estimate that BAT could clear about 1.2 mL of plasma from BCAA per minute, while skeletal muscle (assuming ∼30 kg muscle mass) could clear about 1.2 L of plasma from BCAA per minute (about 1,000-fold higher from BAT). The metabolic fate of BCAA in BAT was beyond the scope of this study, but BCAAs in BAT can be used for oxidation and TCA cycle anaplerosis or for the synthesis of intracellular lipids (TCA cycle cataplerosis).^26^

This study also provides insights on the potential clinical significance of the BCAA clearance by BAT in the adiposity and metabolic health. Specifically, we report that the ^18^F-fluciclovine net uptake rate in supraclavicular adipose tissue gradually decreases with adiposity, and it is inversely correlated with BMI. The correlation between BCAA uptake in supraclavicular adipose tissue and the radiodensity of the tissue—an index of the tissue lipid content^19^—further corroborates the relationship between BCAA clearance rate in BAT and lipid accumulation. BMI was not correlated with ^18^F-fluciclovine delivery rate *K*_*1*_ (index of tissue perfusion) in the supraclavicular adipose tissue, but it was correlated with ^18^F-fluciclovine delivery rate in the abdominal adipose tissue, as it has been previously reported.^27^^,^^28^ These results suggest that the lower ^18^F-fluciclovine net uptake rate in supraclavicular adipose tissue in people with high BMI is not attributable to an adiposity-related lower tissue perfusion. Further, we report that participants with diagnosis of T2D have lower BCAA clearance rate in supraclavicular adipose tissue compared to those without diagnosis of T2D, suggesting that BCAA clearance rate in supraclavicular adipose tissue can be a marker of metabolic health, as it has been suggested for glucose.^29^ Our findings are consistent with data from studies in rodents. Specifically, Yoneshiro et al. have previously reported that BCAA clearance in BAT is implicated in the development of adiposity and insulin resistance,^10^ while Samms et al. have recently reported that tirzepatide (a dual glucose-dependent insulinotropic polypeptide and glucagon-like peptide-1 receptor agonist known to improve glycemia leading to weight loss) also increases BCAA catabolism in BAT. The exact mechanism underlying the link between BCAA clearance in BAT and metabolic health remains to be clarified in light of the recent findings suggesting that leucine, isoleucine, and valine appear to have distinct metabolic roles in the regulation of metabolic processes relevant to the pathogenesis of T2D.^30^^,^^31^^,^^32^

Our findings also indicate that the subcutaneous upper chest adipose tissue depot has a distinctive phenotype in terms of BCAA kinetics and radiodensity compared to the supraclavicular and abdominal adipose tissue depots. Specifically, subcutaneous upper chest adipose tissue has an intermediate phenotype between the subcutaneous abdominal and supraclavicular adipose tissue depot in terms of SUV_mean_, *K*_*1*_ (index of tissue perfusion) and *K*_*i*_ (net uptake rate) for ^18^F-fluciclovine. We also found that differences in tissue perfusion may explain the higher net tissue uptake rate *K*_*i*_ for ^18^F-fluciclovine reported in supraclavicular adipose tissue compared to upper chest adipose tissue, as the *k3* flux rate from interstitial to intracellular compartment for upper chest and supraclavicular adipose tissue depot were similar. The net uptake rate *Ki* for ^18^F-fluciclovine was also correlated with BMI, as it was in the supraclavicular adipose tissue. Upper chest adipose tissue radiodensity was higher than the subcutaneous abdominal adipose tissue depot, but not the supraclavicular adipose tissue depot. These findings provide insights into the heterogeneity of the adipose tissue organ^33^ in people and suggest that different metabolic characteristics assessed in one adipose tissue depot do not necessarily extrapolate to the rest of the adipose tissue depots. Consistent with these results, Cypress et al. have previously reported on the variability in gene expression, differentiation capacity, and oxygen consumption among various adipose tissue depots in human neck.^34^ Our results suggest that this may be true for other upper body adipose tissue depots, and, thus, further research is needed to characterize the heterogeneity between the different adipose tissue depots in humans.

In summary, we provided proof-of-concept *in vivo* functional and transcriptional evidence supporting the notion that supraclavicular adipose tissue—the primary BAT depot in humans—has a higher capacity for BCAA clearance rate in people compared to other subcutaneous adipose tissue depots. In addition, our findings support that supraclavicular adipose tissue BCAA net uptake in people constitutes a biomarker for lower adiposity and improved metabolic health. Further research is needed to better understand the contribution of BAT and the thermogenic adipocytes in adipose tissue depots in whole-body BCAA metabolism and its suggested role in the prevention of the obesity-related metabolic complications.

### Limitations of the study

This study provides functional evidence of the ability of the supraclavicular adipose tissue depot to take up BCAA in people. However, it has limitations. ^18^F-fluciclovine is a leucine analog that is transported in the cells via transporters (i.e., L-type amino acid transporter 1 and the alanine serine cysteine transporter 2^35^^,^^36^) that are not exclusive to leucine. This suggests that ^18^F-fluciclovine kinetics may potentially represent the kinetics of other amino acids as well. In contrast to leucine, ^18^F-fluciclovine is not metabolized intracellularly. Additionally, ^18^F-fluciclovine was originally developed as a cancer diagnostic imaging agent and its metabolism has been mostly, but not exclusively, investigated in the context of cancer using preclinical models.^37^ The analysis of ^18^F-fluciclovine kinetics was conducted in older men with prostate cancer receiving androgen deprivation therapy, and we cannot determine if the same results apply to healthy and/or younger men and women. BAT activity is known to decline with increasing age,^38^^,^^39^^,^^40^ and we expect that younger adults may have a higher net uptake for ^18^F-fluciclovine. The effect of androgen deprivation treatment on BAT metabolism is currently unclear as it has not been investigated in people and results from preclinical models have been inconsistent.^41^^,^^42^ Considering that the results of the kinetics analysis are consistent with results of the gene expression analysis in samples collected from healthy adults, the directionality of the relationship reported in the kinetics analysis is bound to apply to healthy adults, but the magnitude may differ. The quantitative assessment performed in ^18^F-fluciclovine imaging data may not accurately represent the maximal contribution of BAT in plasma BCAA clearance rate as the tracer injection and PET-CT imaging were performed during room temperature conditions (∼20-21°C), regardless the season, with full clothing, while cold stimulation has been reported to increase ^18^F-fluciclovine in rodent BAT.^10^ Future studies, including cold stimulation, are needed to assess the role of BAT in BCAA metabolism in young lean individuals who are more likely to have high amount of active BAT.

## STAR★Methods

### Key resources table

**Table:** 

REAGENT or RESOURCE	SOURCE	IDENTIFIER
**Reagent**		

^18^F-fluciclovine	PETNET Solutions, Sacramento, CA, USA	NA

**Software and algorithms**		

SPSS version 29	IBM Corporation	NA
Graphpad Prism version 10	Graph Pad Software	NA

**Other**		

total-body PET-CT scanner (uEXPLORER)	United Imaging Healthcare	NA

### Resources availability

#### Lead Contact

Further information and requests for resources and reagents should be directed to and will be fulfilled by the Lead Contact, Maria Chondronikola (mc2425@medschl.cam.ac.uk).

#### Materials availability

This study did not generate new unique reagents.

#### Data and code availability

• All data reported in this paper will be shared by the lead contact upon reasonable request.
• This paper does not report additional code.
• Any additional information required to reanalyze the data reported in this work paper is available from the lead contact upon reasonable request.

### Experimental model and study participant details

#### Participants

This is a retrospective review of institutional review board-approved registry-type single-institution study. All patients provided their informed consent. Eligible patients (*n* = 36) were adult males with histopathologically proven prostate adenocarcinoma and documented biochemical recurrence after primary treatment with radical prostatectomy (Table 1). The definition of the American Urological Association was used to establish evidence of biochemical recurrence (i.e., prostate-specific antigen concentration of ≥0.2 ng/mL, with a second confirmatory level detected at 0.2 ng/mL or more).^43^ All patients underwent ^18^F-fluciclvine total-body PET-CT scanning as part of their standard-of-care evaluation for biochemical recurrence of prostate cancer (BCRPC). Considering the participants were older adults and BAT volume/activity declines with increasing age,^38^^,^^39^^,^^40^ the findings of this investigation may underestimate the contribution of BAT in BCAA uptake. The potential effect of sex/gender, ancestry/race/ethnicity and clinical condition on the outcomes of this proof-of-concept study is currently unclear.

#### Study approvals

The study was approved by the Institutional Review Boards of the University of California Davis, the University of Texas Medical Branch in Galveston, the Washington University School of Medicine in St. Louis, MO. Written informed consent was obtained from all participants before their participation in this study.

### Method details

#### Total-body dynamic PET-CT imaging

All participants underwent list-mode dynamic single-bed total-body PET-CT acquisition for 25 min in the supine positions with hands above the head. The scan started immediately before the injection of 312 ± 10 (range 287–347) MBq of ^18^F-fluciclovine on a total-body PET-CT scanner (uEXPLORER, United Imaging Healthcare). The scanner has an axial field of view of 194 cm, PET spatial resolution of ∼3.0 mm in full-width at half-maximum near the center of axial field-of-view, and an 80-detector row CT with a minimum slice thickness of 0.5 mm.^44^ Prior to the acquisition of dynamic PET data, a low-dose CT (tube current: ∼50 mA, tube voltage: 140 kVp) was acquired for attenuation correction and anatomical localization. The tube current was automatically modulated by the scanner manufacturer’s algorithm. The PET data were reconstructed into multiple total-body temporal series, including 32 frames (12 × 5s, 3 × 10s, 3 × 30s, 6 × 60s, and 8 × 120s), which were used for kinetic analysis, and a single 10-min frame between the 4^th^ min post-injection till 14^th^ min, which was used for assessment of mean standardized uptake value (SUV_mean_, g/mL) and visualization. This time window was selected to avoid early phase with high vascular tracer content and to avoid relatively later frames where skeletal muscles tend to demonstrate increasing tracer uptake. All reconstructions were performed using the manufacturer-provided software, employing 3D ordered subset expectation maximization (OSEM) algorithm, with 4 iterations and 20 subsets into a 256 × 256 matrix, rendering an isotropic voxel size of 2.344 mm. All standard corrections were applied (scatter, randoms, dead-time and normalization) without point-spread function (PSF) modeling. No post-reconstruction smoothing was applied. CT images were reconstructed using the manufacturer-provided iterative method with a slice thickness of 2.344 mm to match those of PET and an in-plane voxel size of ∼0.49 × 0.49 mm.

#### ^18^F-fluciclovine-PET-CT imaging analysis

The reconstructed PET and CT images were transferred to a standard analysis workstation running LifeX software (versions 7.2).^45^ PET and CT registration was confirmed visually and any misregistration was corrected if needed. Three volumes of interest (VOIs) were drawn on the fused PET-CT using the brush tool. First VOI was drawn on the left and right supraclavicular areas immediately at the root of the neck corresponding to the known site of BAT in adult human, second VOI on the subcutaneous adipose tissue in the proximal arm/upper chest and back of the shoulder regions (upper chest adipose tissue), and the third VOI on the subcutaneous abdominal adipose tissue at the level of third lumbar vertebra. To ensure adequate circumferential representation, subcutaneous VOIs were placed to sample left and right parts on consistent anatomical regions across subjects as possible (two for supraclavicular, three for upper chest, and four for subcutaneous abdominal). VOIs were at least 5.0 mL in volume. Within each VOI, two sequential sets of thresholds were applied: first on the CT images to include only adipose tissue densities (−190 to – 30 Hounsfield Unit [HU]) then on PET images to exclude any potential spill-over from adjacent skeletal muscles (and other soft tissues with high activity). The study personnel that drew the VOI were blinded to the hypothesis of this investigation.

#### Adipose tissue biopsies from healthy participants and gene expression analysis

Supraclavicular and abdominal adipose tissue samples were collected from a group of adults with overweight/obesity who participated in clinical trials aiming to examine the role of human BAT in metabolic function (Clinical Trials.gov NCT02786251 and NCT01791114). The entire cohort included 23 participants with supraclavicular adipose tissue samples and 12 of them also had matched abdominal adipose tissue biopsies. Table S1 summarizes the demographic and anthropometric characteristics of participants that provided adipose tissue biopsies. Each participant performed a 6-h exposure to mild cold followed by a 2-deoxy-2-[^18^F]-fluoro-glucose PET-CT scan and a supraclavicular adipose tissue biopsy.^21^^,^^22^^,^^46^^,^^47^ An abdominal subcutaneous adipose tissue biopsy was collected from the same participant immediately after the supraclavicular adipose tissue biopsy or 1–4 weeks apart und the same environmental and fasting conditions. During cold exposure, participants wore shorts, a sports bra (females only), a T-shirt/hospital gown and a liquid-conditioned garments (ThermoWrap, Belmont Medical Technologies, Billerica, MA or Polar Products Inc, Stow, OH). The room and cooling equipment temperature were initially set to 22°C and decreased by 1°C–2°C in intervals of 30–60 min until shivering was induced. Immediately upon shivering, cooling equipment temperature was increased by 1°C–2°C until shivering stopped. BAT volume was quantified using the BARCIST 1.0 recommendations.^25^ All tissue samples were rinsed with ice-cold saline immediately after collection, cleaned off connective tissue and blood and immediately frozen in liquid nitrogen. Approximately 100 mg of adipose tissue was used for extraction of RNA using RNeasy Mini Kit (74104; Qiagen, Chatsworth, California) including an on-column DNAse digestion step. The expression of the various genes involved in BCAA metabolism (*BCAT1/2, BCKDHB and SLC25A44*) *and UCP1*, relative to the housekeeping control gene ribosomal protein lateral stalk subunit P0 (*RPLP0*), was determined by using an ABI 7500 Real-Time PCR System (Invitrogen, Carlsbad, California) and Fast SYBR Green Master Mix (4385618, Invitrogen). The primers used are shown in Table S2.

### Quantification and statistical analysis

#### Tracer kinetic modeling

An irreversible two-tissue (2T) compartmental model,^48^^,^^49^ as illustrated in Figure S1, was used to describe the kinetics of ^18^F-fluciclovine PET data. $C_{p}$ is the tracer concentration in plasma. The tracer enters and exits the free-state compartment ($C_{f}$) with the rate of $K_{1}\left(\text{mL}/\min/cm^{3}\right)$ and $k_{2}\;\left(\min^{-1}\right)$. $k_{3}\;\left(\min^{-1}\right)$ is the rate constant of the tracer from $C_{f}$ to the contained compartment $C_{c}$ which may represent the intracellular space.^49^ This irreversible model assumes that there is no exit process from the intracellular space (i.e., $k_{4}=0$). The total activity that can be measured by PET is the sum of different compartments:

$$C_{T}\left(t\right)=v_{b}C_{b}\left(t\right)+\left(1-v_{b}\right)\left(C_{f}\left(t\right)+C_{c}\left(t\right)\right)$$

where $v_{b}\left(\text{mL}/\text{mL}\right)$ is the fractional blood volume. $C_{b}\left(t\right)$ represents the tracer concentration in the whole blood. All the kinetic parameters were estimated by fitting a measured tissue time activity curve (TAC) following the approach we have previously described elsewhere.^50^ An image-derived TAC in the ascending aorta was used as the plasma input function. The macro-parameter of interest is the net uptake rate $K_{i}=\frac{K_{1}k_{3}}{k_{2}+k_{3}}$ in the unit of $\text{mL}/\min/cm^{3}$, which reflects the clearance of ^18^F-fluciclovine from blood to tissue.

#### Statistical analysis

Results are presented as means ± SD or SEM for normally distributed data and as median and interquartile range for skewed data. Tissue differences between paired observations were assessed by using repeated-measures ANOVA (for normally distributed data) with Tukey correction for multiple comparisons and the Friedman test (for skewed data) with Dunn correction for multiple comparisons. Group differences between independent observations were assessed using one-way ANOVA with Tukey correction for multiple comparisons. Within and between subject differences in gene expression were assessed by using the Wilcoxon signed-rank test and the Mann Whitney test respectively. Spearman’s rho was used to evaluate the correlation between the different variables of interest. Statistical analyses were performed by using GraphPad v9 (GraphPad Software, San Diego, CA). All statistical tests assumed a 95% level of confidence as significant.

#### Sample size estimation and statistical power

Based on our preliminary data, we estimated that at minimum 8 subjects in each group would be needed to detect paired differences between supraclavicular and subcutaneous abdominal adipose tissue SUV_mean_ for ^18^F-fluciclovine equal to 0.2 g/mL using a 2-sided test with a power of 0.95 and an α value of 0.05. These computations were performed using G∗Power 3.1.9.6.^51^

### Additional resources

Clinical trial registration number NCT02786251, clinicaltrials.gov (https://clinicaltrials.gov/study/NCT02786251) and NCT01791114, clinicaltrials.gov (https://clinicaltrials.gov/study/NCT01791114).
