# Supplementary material for: The role of brown adipose tissue in branched-chain amino acid clearance in people
Source: iScience. 2024 Jul 20;27(8):110559. doi: 10.1016/j.isci.2024.110559 (PMC11340589; doi:10.1016/j.isci.2024.110559)
Supplement: Document S1. Figures S1–S3, Tables S1, and S2 [file mmc1.pdf]

iScience, Volume *27*

Supplemental information

The role of brown adipose tissue

in branched-chain amino acid clearance in people

Yasser G. Abdelhafez, Guobao Wang, Siqi Li, Vanessa Pellegrinelli, Abhijit J. Chaudhari, Anthony Ramirez, Fatma Sen, Antonio Vidal-Puig, Labros S. Sidossis, Samuel Klein, Ramsey D. Badawi, and Maria Chondronikola

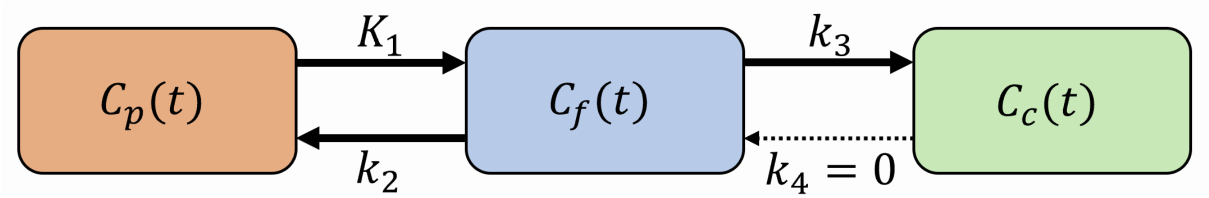

Figure S1. Illustration of the irreversible two-tissue compartmental model used for analyzing dynamic ^{18}F-fluciclovine positron emission tomography data. C_p: tracer concentration in plasma; C_f: free-state (i.e., interstitial space) compartment; C_c: intracellular space compartment. K_1 and $k_{2\text{-}4}$: flux rates between the different compartments. The parameter K_i (net uptake rate) was calculated as follows $K_i = \frac{K_1 k_3}{k_2+k_3}$ in the unit of mL/min/cm^3 and reflects the clearance of ^{18}F-fluciclovine from blood to tissue. Related to Figure 3.

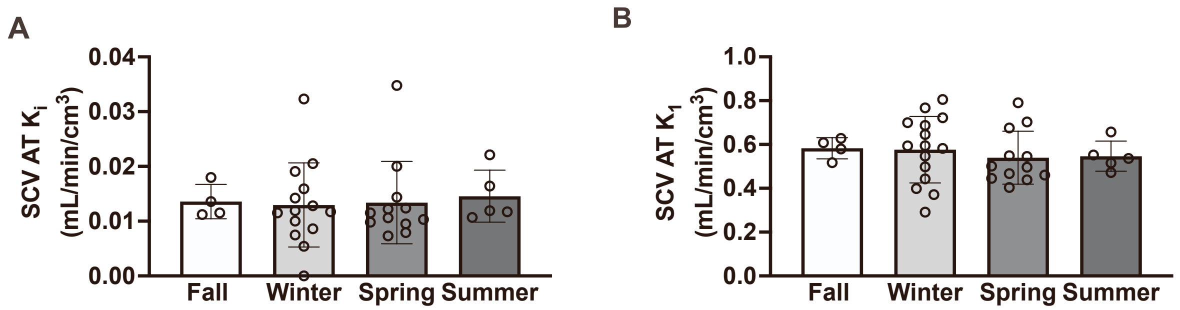

Figure S2. Seasonal variability and ^{18}F-fluciclovine uptake in supraclavicular adipose tissue (n = 35-36). Data presented at mean and SD. AT: adipose tissue, HU: Hounsfield Units, K_i: net uptake rate reflecting cleareance of ^{18}F-fluciclovine from blood to tissue, SCV: supraclavicular, SUV_{mean}: mean standardized uptake value. Related to Figure 3.

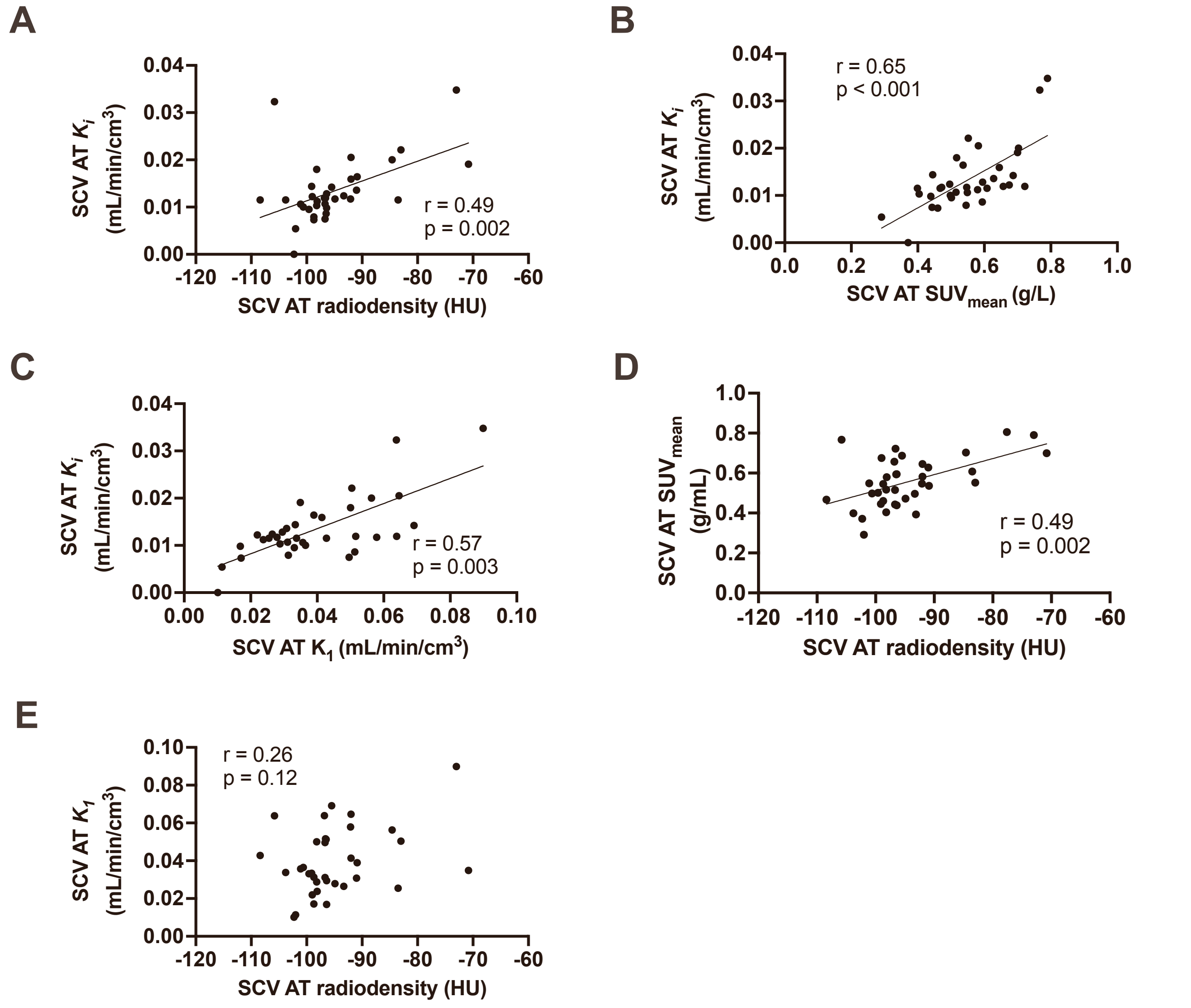

Figure S3. Inter-relationships among supraclavicular adipose tissue radiodensity and ^{18}F-fluciclovine kinetics (n = 35-36). Spearman's rho was used to evaluate the correlation between the different variables of interest. AT: adipose tissue, HU: Hounsfield Units, K_1: ^{18}F-fluciclovine tracer delivery rate, partially reflecting tissue perfusion**,** K_i: net uptake rate reflecting cleareance of ^{18}F-fluciclovine from blood to tissue, SCV: supraclavicular. Related to Figure 3.

Parameters	All<br>n = 23	LBAT<br>n = 7	HBAT<br>n = 16	Participants with paired biopsies<br>n = 12
**Age (years)**	41 ± 12	47 ± 14	38 ± 11	38 ± 12
**Sex (females/males)**	17/6	4/3	13/3	10/2
**Race (White/ Black/Asian)**	20/2/1	5/1/1	15/1/0	12/0/0
**Height (cm)**	169 ± 8	171 ± 5	169 ± 9	166 ± 7
**Weight (kg)**	90 ± 13	90 ± 13	90 ± 14	86.8 ± 11.3
**BMI (kg/m^2)**	31 ± 3	31 ± 3	32 ± 3	32 ± 2
**Body fat (%)**	42 ± 6	41 ± 3	43 ± 7	44 ± 3

Table S1. Demographic and anthropometric characteristics of healthy participants that provided adipose tissue biopsies. Data are means ± SD. HBAT: group of participants with brown adipose tissue volume equal to or more than 20 ml; LBAT: group of participants with brown adipose tissue volume less than 20 ml. Related to Table 1 and Figure 5.

Gene	Accession No.	Forward (F) and Reverse (R) Primer
BCAT1	NM_005504.7	F: CCGACGGAACAATGAAGGATTG<br>R: GTCCCCACCACCTCTTTTGA
BCAT2	NM_001190.4	F: ACAGTTACGCGCCGCA<br>R: CAGGTCTGCAGCCTTGAAAC
BCKDHB	NM_183050.4	F: CCGGGAGTACGGGCAAAC<br>R: TGACCGCAATTCCGATTCCA
RPLP0	NM_001002	F: GTGATGTGCAGCTGATCAAGACT<br>R: GATGACCAGCCCAAAGGAGA
SLC25A44	NM_001286184.2	F: GCTGGGAACGACGGATAGAC<br>R: GGTGCCTGAAGACCTTCTCA
UCP1	NM_021833	F: AGGTCCAAGGTGAATGCCC<br>R: TTACCACAGCGGTGATTGTTC

Table S2. Sequence of primers used for reverse transcription polymerase chain reaction. Related to Figure 5.
